# Developing a National Mental Health Policy: A Case Study from Uganda

**DOI:** 10.1371/journal.pmed.1001319

**Published:** 2012-10-02

**Authors:** Joshua Ssebunnya, Fred Kigozi, Sheila Ndyanabangi

**Affiliations:** 1 Butabika National Referral and Teaching Mental Hospital, Kampala, Uganda; 2 Ministry of Health, Kampala, Uganda

## Abstract

As one article in an ongoing series on Global Mental Health Practice, Joshua Ssebunnya and colleagues provide a case study from Uganda that describes their work developing a national mental health policy.

**Summary Points**

- Mental health policy development is an iterative process that requires wide stakeholder consultations and during which the policy content is revised several times before arriving at the final policy document.
- A small, multidisciplinary drafting committee can increase the efficiency of the process and enrich the content of the draft policy. Comparing and borrowing from policies of other countries of similar socio-economic context can be helpful during the policy development process.
- Here we describe the process of development and adoption of a national policy on mental health in Uganda. This work is based on a study undertaken by the Mental Health and Poverty Project (MHaPP), a research consortium investigating the policy-based, legal, and planning interventions required to break the cycle of poverty and mental illness in low- and middle-income countries.

*This case study is part of the* PLOS Medicine *series on Global Mental Health Practice*.

## Introduction

Mental disorders are a significant public health issue due to their high prevalence and considerable contribution to the global disease burden. The 2001 Global Burden of Disease (GBD) study ranked unipolar depressive disorders as the third leading cause of disease burden, rising to first place for high- and middle-income countries [1].

Evidence suggests that a range of interventions are effective in treating and preventing mental disorders in low- and middle-income countries (LMICs) [2],[3]. However, mental health is not given the priority it deserves in most of these countries [4], resulting in a significant gap between the level of mental health needs and the availability of quality services to appropriately address these needs. In LMICs, especially those in Africa, an estimated 76%–99% of people with serious mental health conditions have no access to the treatment they need [5],[6].

Mental health policies and plans are essential tools for coordinating all mental health services. Without such coordination, mental disorders are likely to be treated in an inefficient and fragmented manner. Policies are more likely to achieve the desired effect when they reflect a clear commitment from governments, are well conceptualized, and are consistent with the existing evidence base and international standards. Furthermore, mental health policies should reflect a broad consensus among key stakeholders [7]. In light of the large global burden of mental illness, there is a clear need for well-developed, articulated, and aggressively implemented national mental health care policies. Such policies should be designed to improve the care of, and reduce the burden on, those individuals with mental health, neurological, and substance use (MNS) disorders.

By 2005, only half of the countries in the WHO African Region had a mental health policy, highlighting the urgency of mental health policy development in Africa. Indeed there has been a clear acceleration of policy development over the last 10 years following the recommendations of the World Health Report 2001, which focused on mental health (http://www.who.int/whr/2001/en/), and the production and dissemination of the WHO Mental Health and Policy Service Guidance package (see Box 1). However, while this trend is promising, very little research has been conducted on mental health policy development and implementation processes, particularly in Africa [3],[5],[8].

**Box 1. WHO Mental Health and Policy Service Guidance Package**

The WHO Mental Health and Policy Service Guidance package aims to provide practical information to help countries improve the mental health of their populations. It provides policy guidance on developing mental health strategies, resource allocation, service provision, and reintegration of patients into community life. There are 14 inter-related modules, such as those on mental health advocacy, financing, and service organization, as well as two WHO checklists to “assess the adequacy and the content of a mental health policy and/or plan as well as the process for developing them.” The modules and checklists are freely available at http://www.who.int/mental_health/policy/essentialpackage1/en/index.html.

This Health in Action article briefly describes the process of development and adoption of a national policy on MNS disorders in Uganda, and describes the content of the adopted policy. The article is based on a study undertaken by the Mental Health and Poverty Project (MHaPP), a research consortium investigating the policy-based, legal, and planning interventions required to break the cycle of poverty and mental illness in LMICs [9].

## Evaluation of the Initial Draft Policy

The study involved a situational analysis of Uganda's mental health system, conducted in 2006–2007, during which a number of gaps in mental health service delivery were identified and the importance of the mental health policy emphasized [10],[11]. During the study, the initial *Draft Mental Health Policy* of Uganda (2000–2005) was evaluated using the WHO mental health policy evaluation checklist, which was developed by the WHO Department of Mental Health and Substance Abuse as a part of its Mental Health Policy and Service Guidance Package [12]. The checklist assesses (a) whether consultative processes have been followed that are likely to lead to the successful adoption and implementation of the policy, and (b) whether the policy content addresses certain critical issues, such as protection of human rights, adoption of evidence-based approaches, and the development of community-based care [13].

The *Draft Mental Health Policy* was found to have several flaws in terms of process and content. These flaws included: (a) Lack of formal approval and official dissemination of the policy; (b) Lack of an evidence base for policy development; (c) Inadequate stakeholder consultation; (d) Absence of a clear vision and unclearly spelt out values and principles underlying the policy; (e) Lack of specificity for financing of the policy.

The general consensus from the evaluation was that the draft mental health policy informally guided the mental health programme and activities nationwide but needed to be revised and finalized into an updated policy, widely acceptable by various stakeholders.

## Process of Developing a New Mental Health Policy

**Figure 1:**
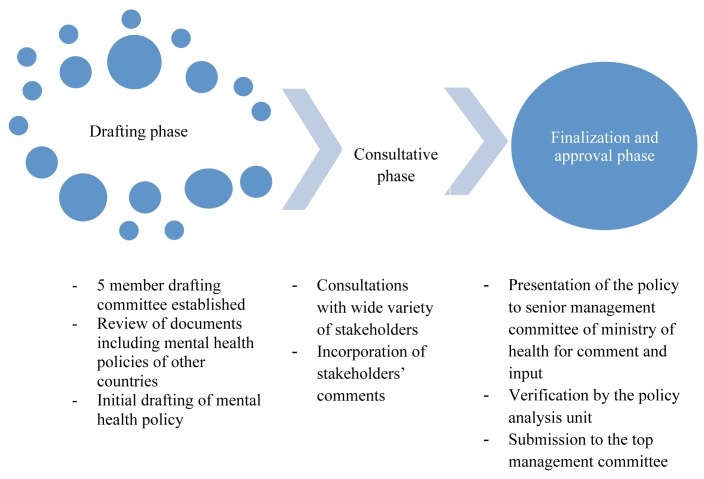
Process of developing a new mental health policy for Uganda.

The Ministry of Health of Uganda embarked on developing a new mental health policy, an exercise conducted over three phases: drafting, consultation, and finalization (see Figure 1).

The drafting phase included setting up a five-member drafting committee made up of mental health professionals and members of civil society. The committee began by reviewing the initial *Draft Mental Health Policy* (2000–2005) and its evaluation report, the mental health policies of four other countries that were accessible (Santa Lucia, Gambia, Nigeria, and Australia), and the report of the 2006–2007 situational analysis of Uganda's mental health system. These documents largely guided the structure and content of the new draft.

Stakeholders from relevant departments, institutions, and civil society were identified and invited to the consultations (Box 2). Individuals were selected on the basis of their expertise and experience. They were briefed on the policy development process and the findings of the 2006–2007 situational analysis. The stakeholders then scrutinized the content of the new draft policy document and discussed ideas they wanted to have included or dropped from the draft. Participants' comments on the content were subjected to discussion during the consultation workshops. Comments that the group deemed relevant were considered for inclusion in the new policy document. The team assessed the feasibility of including these emerging ideas in the new policy document, borrowing from the policy documents of other countries to see how best to present such ideas.

**Box 2. Stakeholders Consulted During Policy Development**

Psychiatrists, psychiatric social workers, psychologists, public health specialists, policy analysts, programme managers within Ministry of Health, representatives of line ministries, human resource development experts, representatives of non-governmental carer and user groups, human rights activists, and private service providers.

As the consultation process was consensus-based, and members had varied views on a number of issues, not all comments were included in the final draft. There were three reasons for excluding comments from the final draft. First, some ideas or comments were more strategic in nature, and so were better placed in the strategic plan rather than in the policy content itself. Second, some of the ideas were perceived to be overly ambitious and were not seen as appropriate given Uganda's current health care context. Third, some of the ideas were inaccurate and would jeopardize the rights of mental health service users, for example the proposal to implement routine surveillance and pick-up of all persons with mental illness, and keeping them in a mental hospital “for the good of society.”

The drafting team subsequently developed a revised version of the policy that was presented to stakeholders during a consensus building workshop for final review. During this exercise, the WHO checklist for mental health policy evaluation was once again used to check for completeness of the revised policy. At this point, stakeholders highlighted the fact that neurological disorders, especially epilepsy, and substance abuse disorders are mostly handled together with mental disorders in the same health facilities, pointing to a need to ensure that these disorders are all accounted for in the same policy. This development was timely, supported by the WHO's adoption of the new term “MNS disorders”—covering mental health, neurological, and substance use disorders together. Consequently, the draft policy was revised further from a National Mental Health Policy to a National Policy on Mental Health, Neurological and Substance Abuse (MNS) Disorders.

The entire process lasted 3 years. In total, there were five stakeholder consultation workshops and two consensus building workshops, in addition to several meetings of the drafting committee.

This revised policy was then presented to the Ministry of Health's Senior Management Committee for its comments and inputs, which were incorporated into the draft, and also to the Ministry's Policy Analysis Unit to make sure that it was is in line with Ministry of Health guidelines and expectations. Finally, the revised policy was sent to the Top Management Committee of the ministry for approval.

## Content of the New Policy

Feedback from various stakeholders suggests that, compared with the old policy, the new mental health policy is more comprehensive, embracing factors that affect recovery such as stigma and poverty. The new policy also has a stronger management framework that enhances public private partnerships and involvement of mental health service users.

As shown in Table 1, the new mental health policy includes a vision statement, a set of eight guiding principles, and six priority areas. The table also summarizes some of the key policy objectives.

**Table 1:** Vision, guiding principles, key priority areas, and selected policy objectives.

Vision	Guiding Principles	Key Priority Areas	Selected Policy Objectives
A population free of MNS disorders	1. Right to health, regardless of sex, race, and creed.	1. Availability and access to quality MNS services to the population	1. To increase availability of, and access to, quality MNS services
	2. Equitable distribution of services	2. Capacity of health workers at all levels of health care to provide MNS services	2. To provide services in a multifaceted and multidisciplinary manner, ensuring the relevant skills mix
	3. Use public–private partnerships in delivering MNS services	3. Strengthening community mobilization for involvement and participation	3. To ensure collaboration with traditional and complementary practitioners in order to provide coordinated service delivery
	4. Community involvement and participation	4. Strengthening Health Management Information System (HMIS), monitoring and evaluation, and research	4. To strengthen the capacity of health workers, at all levels, to provide MNS services
	5. Services of the highest standard possible according to current scientific knowledge and available resources	5. Advocacy and fundraising for MNS services	5. To promote and strengthen the involvement and participation of all stakeholders in NMS services
	6. Ethical code of conduct and promotion of integrity	6. Partnership and collaboration for MNS care services	6. To strengthen community involvement and participation
	7. Efficiency in the provision of services		7. To encourage cooperation between the services and programs needed to enable people with MNS problems to participate fully in community life
	8. Gender sensitivity in all program areas		8. To mobilize financial resources for MNS service delivery by ensuring equity, efficiency, transparency and accountability
			9. To ensure the relevant laws and regulations concerning MNS issues in Uganda are developed and enforced
			10. To harness scientific knowledge through research for evidence-based policy and decision making

Later analysis of the revised MNS draft policy by a group of stakeholders using the WHO checklist found the following strengths: (a) The values and associated principles in the policy promote human rights, social inclusion, evidence-based practice, inter-sectoral collaboration, and equity with physical health care. (b) The policy clearly shows how funding will be used, emphasizing equitable funding for mental health and physical health. (c) The policy highlights the need for updated mental health legislation that upholds the rights of people with mental health problems. (d) Integration of mental health services into general health services is emphasized. (e) Promotion, prevention, and rehabilitation are comprehensively addressed in the policy. (f) The policy addresses improving the availability and accessibility of essential psychotropic medicines and strengthening the capacity of health workers at all levels to provide mental health service through training and recruitment. (g) The policy advocates for consumers and for community participation and involvement in care. (h) There is satisfactory focus on quality improvement, as the policy makes a commitment to providing evidence-based interventions and includes a process for measuring and improving quality of services. (i) The policy promotes intra-sectoral and inter-sectoral collaboration between non-governmental organizations and government departments, and also supports the strengthening of public–private partnerships for MNS service delivery. (j) Finally, the policy addressed the need for research and evaluation to improve services.

## Conclusion and Lessons Learned

Successful mental health policy development can be a lengthy iterative process that requires high level mandate, leadership, and commitment. The process becomes easier if preceded by a situational analysis and other collateral evidence highlighting the need for the policy, and should be informed by wide stakeholder consultation. Importantly, attempting to consider and include all comments and concerns to the stakeholders' satisfaction can be a hurdle that makes the process unnecessarily long. For a smooth process, it is necessary to carefully identify and select the stakeholders to be involved.

## Abbreviations

LMIC: low- and middle-income country
MHaPP: Mental Health and Poverty Project
MNS: mental health, neurological, and substance use
